# Genotypic Diversity Is Independent of Pathogenicity in Colombian Strains of *Cryptococcus neoformans* and *Cryptococcus gattii* in *Galleria mellonella*

**DOI:** 10.3390/jof4030082

**Published:** 2018-07-05

**Authors:** Norida Velez, Maira Alvarado, Claudia Marcela Parra-Giraldo, Zilpa Adriana Sánchez-Quitian, Patricia Escandón, Elizabeth Castañeda

**Affiliations:** 1 Grupo de Microbiología, Instituto Nacional de Salud, Bogotá 110931, Colombia; noridavelezc@gmail.com (N.V.); maira8822@gmail.com (M.A.); ecastaneda21@gmail.com (E.C.); 2 Unidad de Investigación en Proteomica y Micosis Humanas, Grupo de Investigacion en Enfermedades Infecciosas, Dpto de Microbiología, Facultad de Ciencias, Pontificia Universidad Javeriana, Bogotá 110231, Colombia; claudia.parra@javeriana.edu.co (C.M.P.-G.); adrbiology@gmail.com (Z.A.S.-Q.)

**Keywords:** *Cryptococcus neoformans*, *Cryptococcus gattii*, pathogenicity, *Galleria mellonella*, multi locus sequence typing (MLST), Colombia

## Abstract

Cryptococcosis is a potentially fatal opportunistic mycosis that affects the lungs and central nervous system. It has been suggested that certain strains of *C. neoformans/C. gattii* may have the potential to be more virulent according to the molecular type. This study aims to investigate the association between virulence in the *G. mellonella* model and genotypic diversity of Colombian clinical and environmental isolates of *C. neoformans/C. gattii*. A total of 33 clinical and 12 environmental isolates were selected according to their geographical origin and sequence types (STs). Pathogenicity was determined using the *G. mellonella* model, and the cell and capsular size before and after inoculation was determined. For *C. neoformans*, virulence in *G. mellonella* revealed that death occurred on average on day 6 (*p* < 0.05) and that ST5C, 6C, 25C and 71C were the most virulent. In *C. gattii*, death occurred at 7.3 days (*p* < 0.05), and ST47C, 58C, 75A and 106C were the most virulent. Capsular size increased for both species after passage in *G. mellonella*. In conclusion, the pathogenicity of *Cryptococcus* strains in the *G. mellonella* invertebrate model is independent of molecular type or pathogenicity factor, even within the same ST, but it is possible to find variable degrees of pathogenicity.

## 1. Introduction

Cryptococcosis is a potentially fatal opportunistic mycosis affecting the lungs and central nervous system [1]. The infection is presumed to be initiated by the inhalation of 4–6 µm fungal propagules present in the environment. Two species cause this disease: *Cryptococcus neoformans* (var. *grubii* and var. *neoformans*), which is widely distributed and mainly affects immunosuppressed individuals, and *Cryptococcus gattii*, which can be found in tropical, subtropical, and temperate regions and affects primarily immunocompetent individuals or patients with certain predisposing risk factors [1,2]. Recently, a new nomenclature has been recommended, naming the isolates as *C. neoformans* species complex and *C. gattii* species complex [3].

The virulence of human pathogens has been studied classically in mammals, the mouse being the most widely used model. In recent years, interest has been shown in the use of alternative non-vertebrate models such as *Galleria mellonella* larva due to the cost and bioethical implications of experimentation with mammalian models [4,5,6]. The characteristics that make *G. mellonella* a good model for fungal pathogenesis are hemocytes, expert phagocytes, and the large number of antimicrobial peptides in its hemolymph [4]. An example of this potential has been the use of this model in the study of virulence in pathogenic fungi such as *C. neoformans/C. gattii* [4,5]. *Candida albicans*, *C. tropicalis*, *Histoplasma capsulatum*, and *Paracoccidioides lutzii*, among others [6,7,8,9]. The cellular and humoral innate immune response of *G. mellonella* to infection resembles the activity exerted by neutrophil macrophages during the innate immune response in mammals [4], which is the most important response to control fungal infection.

Similar responses have been recorded during *C. gattii* infection in both murine and *G. mellonella* models [10], suggesting that this model can be used for the study of morphological changes during infection, including factors such as capsule size, which is an important virulence factor for the survival of the fungus in the host. The use of this model may help to understand the mechanisms that result in the development of infection caused by *C. neoformans* and *C. gattii* [4,6,7,10].

To study the epidemiology of *C. neoformans* and *C. gattii*, previous studies have implemented a wide variety of molecular biology techniques, such as polymerase chain reaction (PCR) fingerprinting [11], restriction fragment length polymorphism (RFLP) of the *PLB1* and *URA5* genes [12,13], and multi locus sequence typing (MLST), in which seven conserved genes (*CAP59, GPD1, LAC1, PLB1, SOD1, URA5* and *IGS1*) are used [14].

Some studies have revealed an association between molecular type and virulence; isolates of the molecular type VGIIa recovered from the outbreak reported on Vancouver Island, British Columbia, were reported to be more virulent in a mouse model than the strains of the minor subtype, VGIIb [15]; in addition, Thompson et al. reported molecular-type specific differences when testing the virulence of *C. gattii* strains in *Drosophila melanogaster* [16]. In this study, we set out to investigate the association between virulence and genotypic diversity of Colombian clinical and environmental isolates of *C. neoformans* and *C. gattii* in the invertebrate model *G. mellonella.*

## 2. Materials and Methods

### 2.1. Isolates

A total of 45 isolates of *C. neoformans* and *C. gattii* recovered in 9 departments in Colombia between 1993 and 2014, stored in the collection of the Microbiology Group of the Instituto Nacional de Salud, were selected. Of these, 33 isolates were of clinical origin (24 *C. neoformans* and 9 *C. gattii*), and 12 isolates were from environmental sources (7 *C. neoformans* and 5 *C. gattii*). Inclusion criteria were source (clinical or environmental), geographical origin and ST. A detailed description of the *C. neoformans* and *C. gattii* strains used in the study is described in Table S1.

For *C. neoformans*, a total of 24 clinical and 7 environmental isolates were used for the analysis. Among the clinical isolates, 79.1% were male; the mean age was 39 years, and HIV/AIDS was diagnosed in 79.1% of cases. The 7 environmental isolates came from plant material (*n* = 6) and bird droppings (*n* = 1). For *C. gattii* isolates, nine were of clinical origin, and five were environmental. Among the clinical isolates, 55.5% were male; the average age was 37 years, with HIV/AIDS reported in 33.3%, cases. Five environmental isolates came from three different tree species.

### 2.2. Macroscopic Morphology

Each isolate was plated onto Sabouraud dextrose agar (SDA) and incubated at 27 °C for 48 h, and an inoculum was adjusted to 3.0 × 10^7^ CFU/mL with a spectrophotometer at a wavelength of 530 nm and a reading of 0.561 absorbance. A dilution of 1:1000 and 100 µL of the inoculum was inoculated onto SDA incubated at 27 °C, and 20 colonies were randomly selected. The morphological characteristics were observed by macroscopic visualization according to the texture (mucoid or non-mucoid) and aspect of the colony (wrinkled and smooth) for seven days [17].

### 2.3. Mating Type Determination

DNA extraction was performed as described by Casali [18]. Mating type was determined using PCR, according to the conditions described by Halliday et al. [19]. PCR primers MFα and MFα2 were used. Products were visualized on a 1.2% agarose gel in 1× buffer (Tris Borate EDTA) at 100 V for 1 h. The interpretation was performed by the amplification of a band at 109 bp corresponding to mating type α and a band of 140 bp corresponding to mating type **a**.

### 2.4. Invertebrate Model Galleria mellonella

The larvae were obtained from a Scientia breeding facility (Cali, Colombia), late-stage larvae (fifth and sixth) with weights between 250 and 330 mg and a length of approximately 2 cm were selected. A group of 20 larvae were used for each of the controls: absolute control; larvae without any manipulation, inoculation control; larvae inoculated with phosphate buffered saline (PBS) to monitor killing due to physical injury, and disinfection control; larvae disinfected with sodium hypochlorite to monitor toxicological effects of disinfections. To compare mortality, we performed three biological replicates, with 20 larvae for each strain evaluated [5,10].

The strains were grown in SDA and incubated for 48 h at 27 °C. Suspensions, adjusted to 1.5 × 10^8^ UFC/mL using a Neubauer chamber, were used to inoculate 20 larvae per isolate, each with 10 µL of inoculum by injection into the last left proto-leg using a 0.5 mL gauge insulin syringe [5,10].

After inoculation, larvae were placed in Petri dishes and incubated in darkness at 37 °C, and the number of dead larvae were recorded daily [10]. A detailed description of the control strains of *C. neoformans* and *C. gattii* used in the study is shown in Table S2.

### 2.5. Cellular and Capsular Size Determination

The cellular and capsular sizes of *C. neoformans* or *C. gattii* were determined pre- and post-inoculation of the larvae with each isolate.

Isolates were cultured in Sabouraud broth for 48 h at 150 rpm at 30 °C; next, a microscopic slide containing one drop of Indian ink and one drop of each strain was prepared and visualized in a Zeizz Axiophot Microscope in a 40× lens. The total cell and capsule area was measured, and the cell area was calculated according to the total area minus the area of the capsule (20 cells were measured for each isolate). A small capsule size was established as a measurement of 0.6 to 2.2 µm; intermediate, 2.3 to 3.3 µm; and large, 3.3 to 4.2 µm [20].

To determine these measurements after inoculation in the larvae, strains were recovered from *G. mellonella* after the survival assay. Each dead larva was macerated and homogenized in 1 mL of 1× PBS and the procedure previously described in preinoculation was performed to measure cell and capsular size [10,20]. A detailed description of the control strains of *C. neoformans* and *C. gattii* used in the study is shown in Table S2.

### 2.6. Statistical Analysis

Data collection was tabulated in the Microsoft Corporation Excel^®^ program; the analysis was performed separately for *C. gattii* and *C. neoformans*. Stata software version 11.0 was used, and numerical variables were developed by means of measures of central tendency, and a Chi square test or Fisher exact test was used for categorical variables, with significance lower than 0.05% and 95% confidence. Survival analysis was performed using the Kaplan–Meier method for the invertebrate model; the analysis of the effect of capsular and cellular change before and after inoculation was performed using the McNemar statistic generated with 95% confidence [21].

## 3. Results

### 3.1. Macroscopic Morphology

With respect to colony morphology, 91.1% of the clinical isolates presented smooth mucoid colonies, three of the remaining isolates showed non-mucoid colonies with smooth borders, and one isolate exhibited a wrinkled border. The mean colony diameter for *C. neoformans* was 4.3 mm, with maximum values of 6.8 mm and minimum values of 3.0 mm; for *C. gattii*, this measure was 4.1 mm, with maximum values of 6.9 mm and minimum values of 2.3 mm (Table S1). For the two species, it was observed that the largest colonies were present in ST25 and ST106 for clinical *C. neoformans* and *C. gattii,* respectively.

### 3.2. Mating Type Determination

All *C. neoformans* isolates were mating type α; for *C. gattii*, 64.3% of isolates were mating type α, and the remaining 35.7% were mating type **a**. Sequence types that grouped clinical and environmental isolates had the same mating types (ST77 and ST93) in *C. neoformans* and ST 25 in *C. gattii* (Table S1).

### 3.3. Invertebrate Model Survival Curves

**Figure 1:**
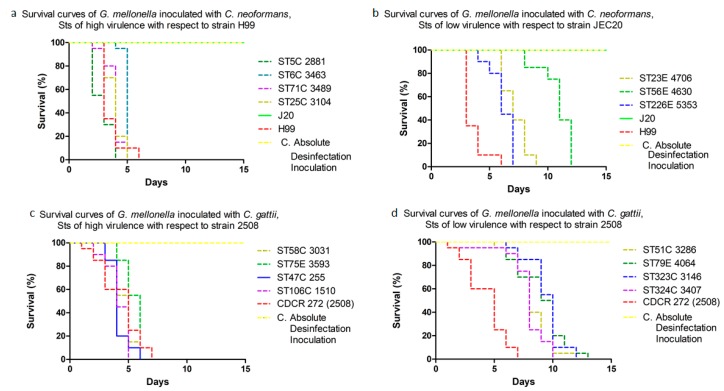
Survival curves of *Galleria mellonella* inoculated with Colombian isolates of the *Cryptococcus neoformans* species complex (**a**,**b**) and *Cryptococcus gattii* species complex (**c**,**d**).

*C. neoformans* virulence in *G. mellonella* revealed that death occurred, on average, on day 6 (*p* < 0.05); ST5C, 6C, 25C and 71C were the most virulent, with a mean survival of 4.5 days, with respect to control strain H99 (Figure 1a,b). For *C. gattii*, larvae death occurred at 7.3 days (*p* < 0.05); ST47C, 58C, 75E and 106C were the most virulent, with a survival of 5.5 days with respect to control strain CDCR-272 (H0058-I-2508) (Figure 1c,d). In contrast, ST 23A, 56A, 226A for *C. neoformans* and the three isolates corresponding to the ST79E for *C. gattii*, showed virulence comparable to the highly virulent strains H99 and CDCR-272, respectively.

**Figure 2:**
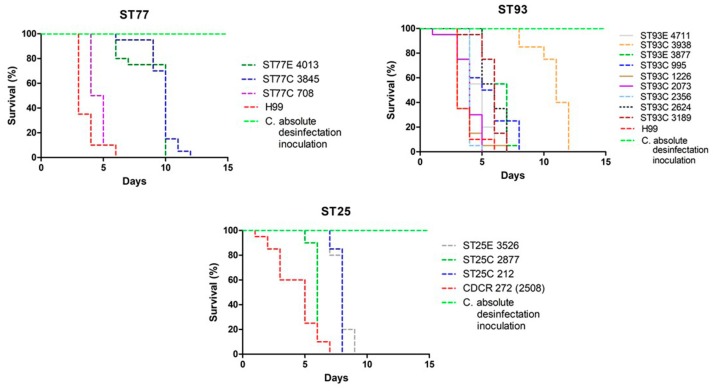
Survival curves of *G. mellonella* inoculated with Colombian isolates of the *C. neoformans* species complex (ST77 and ST93) and *C. gattii* species complex (ST25).

ST5C and ST106C for *C. neoformans* and *C. gattii,* respectively, were responsible for the higher mortality of larvae in less time. *C. neoformans* ST77 and ST93, and *C. gattii* ST25, assigned to clinical and environmental isolates, had a similar degree of virulence in the invertebrate model (Figure 2) (Figures S1 and S2).

### 3.4. Cellular and Capsular Size Determination Pre- and Post-Inoculation

Among the virulence factors of *C. neoformans* and *C. gattii* evaluated, differences were found in capsular and cellular size (pre-inoculation and post-inoculation). The mean total cell size for *C. neoformans* was 5.66 μm and for *C. gattii* was 4.28 μm. A total of 66.6% of the isolates evaluated presented medium size capsules for the two species, and it was observed that the largest capsules in pre-inoculation were presented in ST93 and ST47 for *C. neoformans* and *C. gattii,* respectively, in isolates of clinical origin with respect to the environmental ones (Table 1, Table S1).

**Figure 3:**
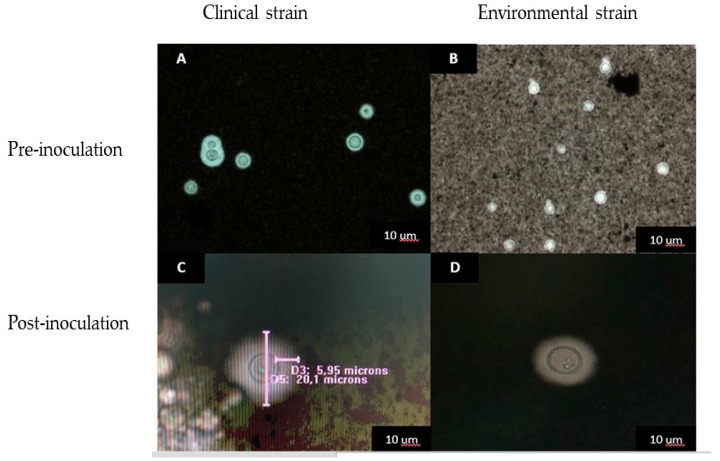
*C. neoformans* capsules with Indian ink (80X). (**A**,**C**) ST93 clinical isolate (H0058-I-2073), pre and post inoculation; (**B**,**D**) ST93 environmental isolate (H0058-I-4711) pre and post inoculation.

The capsular size evaluated after inoculation in the invertebrate model increased for both species, as follows: for *C. neoformans,* 1.57 µm post-inoculation with respect to a 0.60 µm pre-inoculation with a change of ≤0.97 µm on average; in *C. gattii*, 1.92 µm post-inoculation and 0.87 µm pre-inoculation with a change of ≤1.1 µm. The cell size increased by 1.97 µm for *C. gattii* and 0.82 µm for *C. neoformans*, with larger cells being observed after recovery from the invertebrate model (Figure 3).

Observing each ST individually, it was evident that *C. neoformans* ST93C and 307C showed an evident change in capsular size; specifically, 2.46 μm on average. In *C. gattii*, ST 25C showed an average change of 3.42 µm (Table S1, Table 2). We found statistically significant differences between capsular size pre- and post-inoculation (*p* < 0.005).

## 4. Discussion

Phenotype traits in *Cryptococcus* spp. have diverse effects on virulence, and changes in the phenotype characteristics allow adaptation to the environment. Variation in colony morphology and principally mucoid colonies have been associated with strain virulence [15,20,21,22], although this virulence factor may be conditioned by phenotypic changes in the initial morphology due to its ability to undergo phenotypic switching in response to environmental conditions Fraser et al. [15] reported this in 2005, when describing isolates from the Vancouver outbreak, in which colony morphologies in 95% were smooth mucoid, characterized by high virulence. The latter relates to the present investigation of colony morphology and differs with texture. Most of the results obtained in the present study agree with what is typically reported worldwide in terms of colony phenotype [15,21,22,23].

Studies of the pathogenesis of microorganisms in invertebrate hosts during the last decade have contributed to knowledge about the mechanisms of pathogenesis and host defense. The *G. mellonella* model has shown correlation in the results obtained from the pathogenicity of microorganisms in the larva with that presented in vertebrate animal models. In this work, it was observed that there is a high variability among the isolates in their pathogenic capacity and that this one is not associated with the origin of the isolate. Several studies agree that mating α is considered a factor associated with a high virulence [23] and, in turn, is frequent in *C. neoformans* clinical and environmental isolates [24]. Our results are evidence that the mating type α was determined in all isolates of *C. neoformans* and in most of the isolates of *C. gattii.*

In the present work, the capsular and cellular size was evaluated, pre-inoculation and post-inoculation, in the invertebrate model; significantly larger capsules were observed after the fungus passed through the larvae for both species, which is related to several pathogenicity studies such as that performed by Firacative et al. [10], who found increased cell and capsular size after inoculation, where the total cell size of *C. gattii* before infection ranged from 5 to 12 microns and after the inoculation varied from 15 to 75 microns [10]; this increase of the capsule size in some isolates agrees with some studies that have revealed the influence of temperature on capsule size, such as that reported by García-Rodas et al., who demonstrated that the magnitude of capsule increase depended on the temperature, being more pronounced at 37 °C than at 30 °C [25].

The morphology of colonies can be smooth, mucoid, or wrinkled, with increased virulence associated with a mucoid or wrinkled phenotype [26,27]. We found smooth and mucoid colonies as the most frequent morphology in clinical and environmental isolates, with the largest colonies in ST 25 and 106, ST 106 being the most virulent in the invertebrate model. However, the cellular and capsular size determined post-inoculation did not show changes, evidencing the same increase in size for different STs. According to the study carried out by Byrnes et al. [28], the strains that showed variant colony morphologies were evaluated by MLST and were identical across all 8 gene loci tested, revealing that different phenotypic variants maintain similar genetic profiles.

However, it was observed that three clinical isolates did not present a significant change in capsular size, which coincides with several investigations when finding small capsules or cells without capsules recovered from clinical cases. Kimura et al. [29], reported a case of pulmonary cryptococcosis due to a non-capsular strain in a patient with hepatocarcinoma. Laurenson et al. [30], reported a case of meningitis without a capsule in an HIV-infected patient. Moser et al. [31], Ro et al. [32], and Harding et al. reported this same phenomenon in a patient with pulmonary blastomycosis [33], and studies by Salkowski and Balish [34] in animal models showed that a capsule is not always necessary for *C. neoformans* and *C. gattii* to cause disease in mice. The studies of Sabiiti et al. [35] showed that easily phagocytosed strains, namely, “high-uptake” strains or hypocapsular, have been associated with central nervous system fungal burden and patient death, in this case, the enhanced laccase activity was an important virulence factor. Bouklas et al. [36] found hypocapsular strains to have significantly enhanced laccase activity and high virulence in mice, but not in *Galleria*. These data reaffirm that the virulence of *Cryptococcus* strains varies greatly, highlighting some important differences between the various infection models.

Among the tested sequence types of isolates from clinical cases and environmental samples, it is possible to say that only in ST77 was there an association found between mortality in the invertebrate model, even though virulence factors presented similar values between the tests; however, with this evidence, it cannot be concluded that STs are related to virulence factors. It is suggested in future investigations to expand the cohort of isolates related to each ST to be evaluated for different virulence factors, as proposed by Beale et al. [37], who genotyped a cohort of 230 clinical cases in South Africa by MLST to conduct a genetic diversity study of *C. neoformans* and sought relationships between the genotype, phenotype and clinical presentation of the disease. The authors conclude that clinical and phenotypic differences were detectable among genetic lineages; however, explaining the complex relationships between genetic diversity, disease presentation and outcome in a host is difficult [37].

In this study, no specific ST strains predominated with high mortality, colony morphology, or cell or capsular size. Although this molecular characterization is a great taxonomic tool, the method did not show any significant clustering or association to permit distinguishing virulent from less virulent strains from clinical and environmental origins. The study carried out by Litvintseva and Mitchell [38] with a genotyping technique based on hybridization with retrotransposon-specific probes, using the intranasal murine model of cryptococcosis to compare the lethality of clinical and environmental strains of serotype A, allows for establishing differences in virulence between isolates that possess identical genotypes, previously determined by the AFLP and MLST [38]. This association between virulence and molecular type was also established in the isolates recovered from the Vancouver outbreak, in which *C. gattii* VGIIa isolates had a higher virulence profile [27].

In summary, these findings demonstrated that pathogenicity of neither *C. neoformans* nor *C. gattii* in the invertebrate model of *G. mellonella* is specifically associated with a specific virulence factor. Several studies have determined that the virulence of a strain is highly variable, not only between different isolates but also between cells of the same strain, demonstrating that different factors can influence virulence, as suggested by Trevijano-Contador N et al., who demonstrated the effect of different conditions on the formation of titan yeast cells [39].

## Figures and Tables

**Table 1:** Phenotypic assays of *C. neoformans* and *C. gattii* clinical and environmental isolates inoculated in the invertebrate virulence model of *G. mellonella*. Association with sequence type (ST).

Species Complex	ST	Strain (HOO58-I-)	Colony Morphology		Cellular and Capsular Size in µm			
			Texture	Appearance	Pre-Inoculation		Post-Inocultion	
					Cellular	Capsular	Cellular	Capsular
*C. neoformans*	Clinical							
	2	3746	Mucoid	Smooth	7.53	0.21	0.52	4.49
	2	3852			5.34	0.48	1.60	6.98
	5	2881			5.57	0.52	1.76	4.58
	6	3463			5.68	0.50	2.88	4.05
	25	3104			5.49	0.75	0.49	6.14
	32	2340	Non-mucoid		5.87	0.48	0.56	5.42
	40	3589	Mucoid		6.20	0.48	1.63	4.40
	63	2503			5.75	0.52	0.87	4.48
	69	3099			5.87	0.45	1.81	5.96
	71	3489			3.30	0.34	1.60	5.22
	77	708			6.71	1.14	1.03	6.40
	77	3845			5.72	0.67	1.60	4.35
	93	2624	Non-mucoid	Wrinkled	6.53	0.45	1.61	11.88
	93	3189	Mucoid	Smooth	6.72	0.59	1.25	11.51
	93	995			5.54	0.59	3.08	9.18
	93	1226			5.99	0.57	0.86	6.39
	93	2073			4.73	1.22	1.02	7.98
	93	2356	Non-mucoid		6.09	0.55	1.10	6.84
	93	3938	Mucoid		6.15	0.48	0.90	4.40
	199	714			5.90	0.93	3.42	9.78
	307	707			5.85	0.92	2.55	7.97
	307	727			5.44	0.83	1.32	8.00
	307	2087			6.19	0.85	3.24	10.33
	307	2274			6.12	0.89	2.25	9.16
	Environmental							
	15	4419	Mucoid	Smooth	5.69	0.65	1.12	6.04
	23	4706			4.29	0.25	1.34	6.80
	56	4630			5.33	0.36	2.56	4.85
	77	4013			5.74	0.67	2.05	4.37
	93	3877			6.12	0.50	0.91	4.23
	93	4711			6.39	0.59	0.91	4.35
	226	5353			1.70	0.29	0.91	4.35
*C. gattii*	Clinical							
	25	212	Non-mucoid	Smooth	4.49	0.68	11.10	2.25
	47	255	Mucoid		5.40	2.88	9.40	1.85
	85	792			3.57	0.66	8.90	2.21
	106	1510			6.93	2.76	7.60	0.90
	25	2877			5.74	0.46	5.53	1.20
	51	3286			5.64	0.33	4.27	2.10
	58	3031			4.98	0.47	6.07	1.10
	323	3146			6.45	0.83	6.69	6.10
	324	3407			4.40	1.39	4.29	1.40
	Environmental							
	25	3526	Mucoid	Smooth	3.08	0.25	5.69	1.20
	75	3593			2.45	0.65	5.15	1.90
	79	4064			1.86	0.29	4.26	1.60
	79	3080			1.89	0.25	4.30	1.60
	79	3874			3.09	0.29	4.28	1.50

Low capsule size (from 0.6 to 2.2 μm), medium capsule size (from 2.3 to 3.3μm) and high capsule size (from 3.3 to 4.2 μm).

**Table 2:** Analysis of pre- and postinoculation survival of *C. neoformans* and *C. gattii* clinical and environmental isolates inoculated in the invertebrate virulence model of *G. mellonella*. Association with sequence type (ST).

Species Complex		Isolates		In *Galleria mellonella*				Mean Survival Days
	ST	Clinical	Environmental	Pre-Inoculation		Post-Inoculation		
				Mean Cellular Size	Mean Capsular Size	Mean Cellular Size	Mean Capsular Size	
				µm				
*C. neoformans*	93	7	2	6.03	0.62	7.42	1.29	3.67
	307	4	0	5.90	0.87	8.87	2.34	3.25
*C. gattii*	25	2	1	4.44	0.46	7.43	1.55	4.00

## Supplementary Materials

The following are available online at http://www.mdpi.com/2309-608X/4/3/82/s1. Table S1: General information of *Cryptococcus neoformans* and *C. gattii* clinical and environmental isolates according to species complex, sequence types (STs), macroscopic morphology, cellular and capsular size determination pre- and post-inoculation, mating type determination and mean survival in *Galleria mellonella*. Table S2: Control strains of *Cryptococcus neoformans* and *C. gattii* used in the study of virulence in the invertebrate model of *Galleria mellonella* and in phenotypic assays. Figure S1: Flowchart of the methodology used. Figure S2: Survival curves of *G. mellonella* inoculated with *C. neoformans* (1a clinical isolates-2a environmental isolates). Survival curves of *G. mellonella* inoculated with *C. gattii* (1b clinical isolates-2b environmental isolates).
