# Supplementary material for: Genotypic Diversity Is Independent of Pathogenicity in Colombian Strains of Cryptococcus neoformans and Cryptococcus gattii in Galleria mellonella
Source: J Fungi (Basel). 2018 Jul 5;4(3):82. doi: 10.3390/jof4030082 (PMC6162678; doi:10.3390/jof4030082)
Supplement: Supplementary file 1 [file jof-04-00082-s001.pdf]

Table S1. General information of *Cryptococcus neoformans* and *C. gattii* clinical and environmental isolates according to species complex, sequence types (STs), macroscopic morphology, cellular and capsular size determination pre- and post-inoculation, mating type determination and mean survival time (days) in *G. mellonella.*

Species complex	ST	Strain (H0058-I-)	Macroscopic morphology			Pre-inoculation		Post-inoculation		Mating type	Mean survival in *G. mellonella* (days)
			Texture	Aspect	Diameter (mm)	Cellular (µm)	Capsular (µm)	Cellular (µm)	Capsular (µm)		
C. neoformans	Clinical										
											Suppl. Fig 1a
	2	3746	Mucoid	Smooth	3.70	7.53	0.21	4.49	0.52	α	5.60
	2	3852			4.10	5.34	0.48	6.98	1.60		6.00
	5	2881			3.10	5.57	0.52	4.58	1.76		2.80
	6	3463			3.20	5.68	0.50	4.05	2.88		4.90
	25	3104			6.90	5.49	0.75	6.14	0.49		3.90
	32	2340	Non-mucoid		3.60	5.87	0.48	5.42	0.56		4.00
	40	3589	Mucoid		3.20	6.20	0.48	4.40	1.63		9.90
	63	2503			5.60	5.75	0.52	4.48	0.87		9.20
	69	3099			3.90	5.87	0.45	5.96	1.81		6.40
	71	3489			3.00	3.30	0.34	5.22	1.60		3.90
	77	708			6.30	6.71	1.14	6.40	1.03		4.60
	77	3845			4.40	5.72	0.67	4.35	1.60		9.70
	93	995			6.00	5.54	0.59	9.18	3.08		5.60
	93	1226			5.40	5.99	0.57	6.39	0.86		3.60
	93	2073			4.40	4.73	1.22	7.98	1.02		3.90
	93	2356	Non-mucoid		3.64	6.09	0.55	6.84	1.10		4.10
	93	2624		Rugose	4.37	6.53	0.45	11.88	1.61		5.90
	93	3189	Mucoid	Smooth	4.07	6.72	0.59	11.51	1.25		5.80
	93	3938			3.79	6.15	0.48	4.40	0.9		10.80
	199	714			5.10	5.9	0.93	9.78	3.42		5.70
	307	707			4.90	5.85	0.92	7.97	2.55		4.10
	307	727			4.30	5.44	0.83	8.00	1.32		6.20
	307	2087			4.20	6.19	0.85	10.33	3.24		5.00
	307	2274			5.90	6.12	0.89	9.16	2.25		4.00
	**Environmental**										
											Suppl. Fig 2a
	15	4419	Mucoid	Smooth	3.10	5.69	0.65	6.04	1.12	α	7.80
	23	4706			3.40	4.29	0.25	6.80	1.34		7.10
	56	4630			4.70	5.33	0.36	4.85	2.56		10.80

	77	4013			3.40	5.74	0.67	4.37	2.05		9.05
	93	3877			4.10	6.12	0.50	4.23	0.91		6.35
	93	4711			4.40	6.39	0.59	4.35	0.91		4.75
	226	5353			3.20	1.70	0.29	4.35	0.91		6.15
C. gattii	**Clinical**										
											Suppl. Fig 1b
	25	212	Non-mucoid	Smooth	4.50	4.49	0.68	11.1	2.25	a	8.00
	25	2877	Mucoid		3.90	5.74	0.46	5.50	1.20		6.00
	47	255			5.40	5.40	2.88	9.40	1.85	α	4.20
	51	3286			4.10	5.64	0.33	4.30	2.10		7.90
	58	3031			4.40	4.98	0.47	6.10	1.10		5.00
	85	792			3.60	3.57	0.66	8.90	2.21		8.00
	106	1510			6.90	6.93	2.76	7.60	0.90		4.30
	323	3146			4.80	6.45	0.83	6.70	6.10	a	9.40
	324	3407			3.50	4.40	1.39	4.30	1.40	α	7.90
	**Environmental**										
											Suppl. Fig 2b
	25	3526	Mucoid	Smooth	3.80	3.08	0.25	5.70	1.20	a	8.00
	75	3593			2.30	2.45	0.65	5.20	1.90		5.40
	79	3080			3.80	1.89	0.25	4.30	1.60	α	10.60
	79	3874			3.20	3.09	0.29	4.30	1.50		9.60
	79	4064			3.90	1.86	0.29	4.30	1.60		8.70

Table S2. Control strains of *Cryptococcus neoformans* and *C. gattii* used in the study of virulence in the invertebrate model of *Galleria mellonella* and in phenotypic assays.

Collection ID	Species	Assay involved	Characteristics (Reference)
JEC20	*C. neoformans* var. *neoformans*	In vivo study<br>Mating type	Low virulence in *G. mellonella* (10)<br>Mating type **a** (17)
JEC21		Mating type	Mating type α (17)
H99	*C. neoformans* var. *grubii*	In vivo study	High virulence in *G. mellonella* (10)
H0058-I-580		Cellular and capsular size	Capsular size 0.59-0.85 μm (15)
H0058-I-755			Capsular size 0.44-0.6 μm (15)
H0058-I-737			Capsular size 0.33-0.85 μm (15)
H0058-I-1580		Colony morphology	Non-mucoid, rough border (15)
H0058-I-1580			Non-mucoid, smooth (15)
H0058-I-1320 /B-3506	*C. gattii*	Cellular and capsular size	Capsular size 0.75-0.95 μm (15)
		Colony morphology	Mucoid, smooth (15)
H0058-I-2508 /CDC2272		In vivo study	High virulence in *G. mellonella*

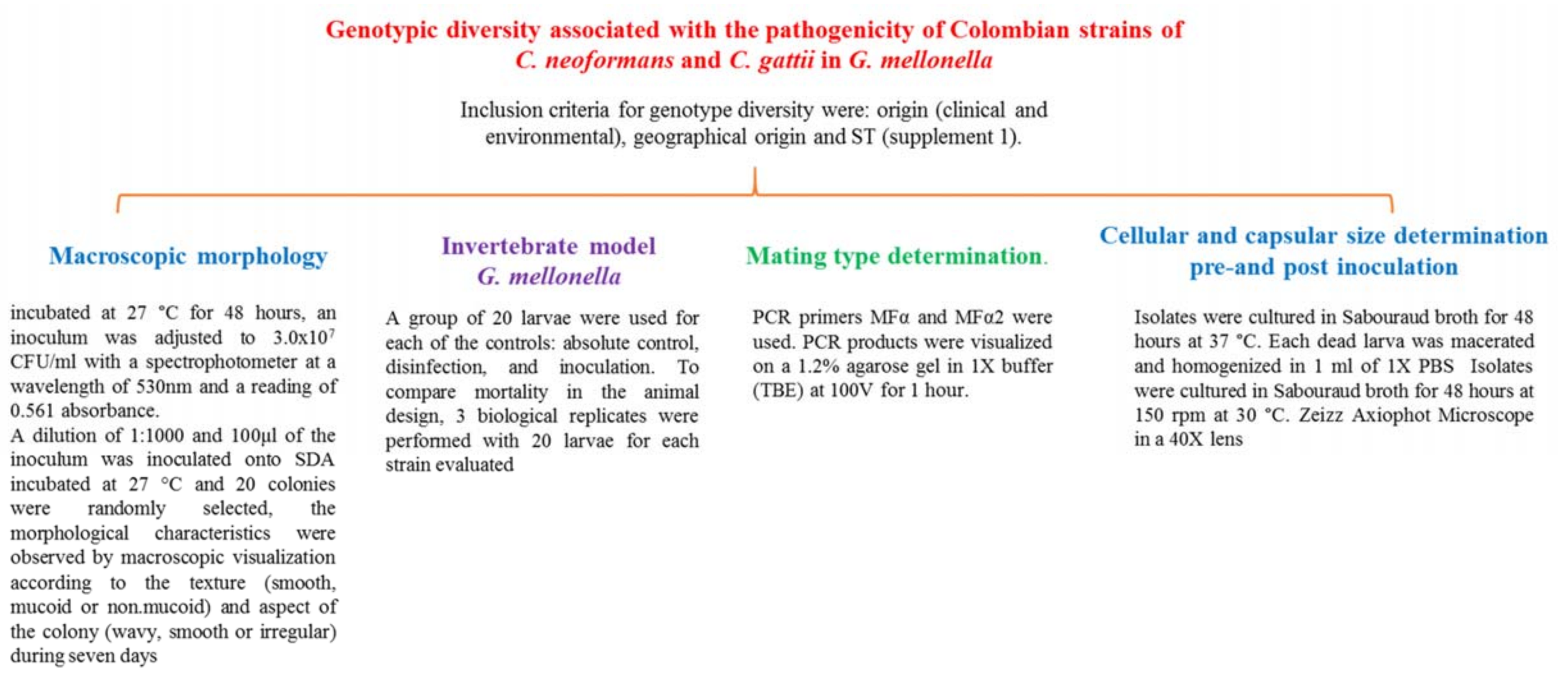

Figure S1. Flowchart of the methodology used. Inclusion criteria, macroscopic morphology, mating type determination, invertebrate model in *Galleria mellonella* and cellular and capsular size determination pre- and post-inoculation.

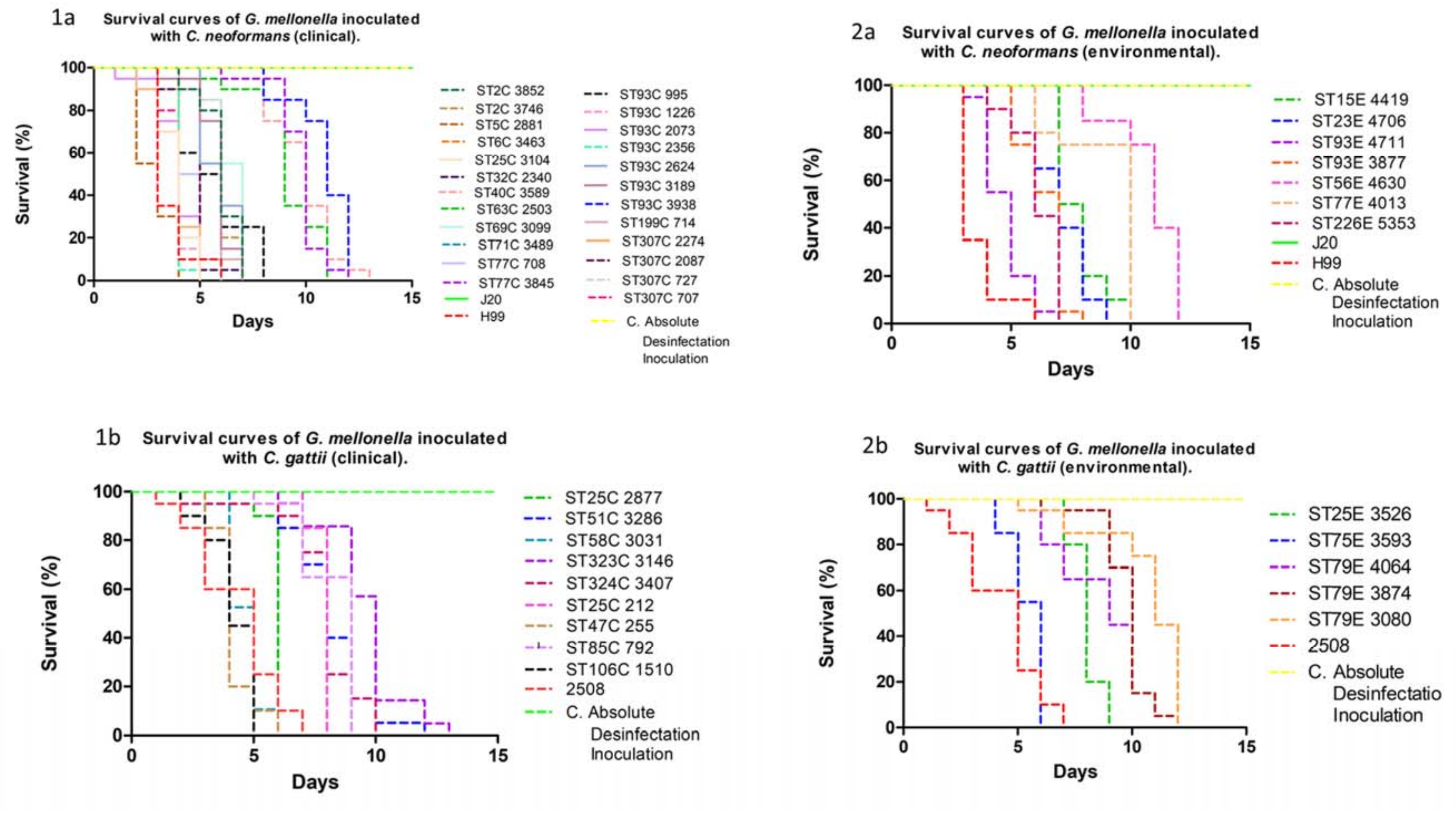

Figure S2. Survival curves of *G. mellonella* inoculated with *C. neoformans* (1a clinical isolates -2a environmental isolates). Survival curves of *G. mellonella* inoculated with *C. gattii* (1b clinical isolates, 2b environmental isolates).
